# London Hospitals with Medical Schools and Their Sites

**Published:** 1903-12-12

**Authors:** 


### HOSPITAL ADMINISTRATION.

### CONSTRUCTION AND ECONOMICS.

### LONDON HOSPITALS WITH MEDICAL SCHOOLS AND THEIR SITES.

A WELL-ATTENDED meeting was held at Charing Cross Hospital on Thursday, December 4th, under the auspices of the Hospitals Association, when a Paper was read by Sir Henry Burdett, K.C.B., on the above subject. The chair was taken by Mr. Thomas Bryant, F.R.C.S., Consulting Surgeon to Guy's Hospital, among those present being Sir Edmund Hay Currie, Mr. Jonathan Hutchinson, F.R.S., Mr. J. Danvers Power, Mr. Timothy Holmes, Mr. Bruce Clarke, Dr. J. H. Morgan, Mr. S. M. Quennell, Mr. Andrew Motion, Colonel Montefiore, and Mr. Sydney Phillips, the Hon. Secretary of the Hospitals Association.

ABSTRACT OF SIR HENRY'S PAPER.

In London the medical schools are, with two exceptions, attached to, and intimately connected with, hospitals, the exceptions being King's College and University College, which are entirely separate bodies, quite independent of the hospitals which bear their names. Only quite recently all the medical schools, with these two exceptions, were within the premises of the hospitals to which they are attached. At Charing Cross this arrangement does not now obtain, the medical school having been removed to a separate site across the street, which is connected with the hospital by a subway under Chandos Street. In none of the London schools will be found such complete and extensive buildings as are to be seen at Edinburgh, Glasgow, or at Owens College, Manchester; and in many of the schools the plans show very particularly that the buildings have been added to from time to time as opportunity or funds would permit. The four great medical schools of the Metropolis are St. Bartholomew's, St. Thomas's, Guy's, and the London. There are also medical schools in connection with St. George's Hospital, Charing Cross Hospital, University College Hospital, Middlesex Hospital, St. Mary's Hospital, the Royal Free Hospital, Westminster Hospital, and King's College Hospital.

THE SITES OF THE EIGHT SMALLER LONDON GENERAL HOSPITALS WITH MEDICAL SCHOOLS.

As I have often maintained, the sites of the London hospitals, taken collectively, represent the best and worst features which could attach to hospitals in densely crowded neighbourhoods. The thing to be aimed at is to have a site of sufficient area to provide at least 300, or, to meet modern hygienic requirements, 450 superficial feet per bed. Each site should be self-contained—that is to say, it should be so placed as to enable the authorities to prevent the hospitals from being deprived of light and air by contiguous buildings. To insure this result, it is desirable that every City hospital site should be entirely surrounded by streets of a reasonable width. Now, applying these tests, we find that, of the eight smaller London general hospitals with medical schools:—

St. George's has a total site area of 67,192 feet, which gives an area of 191 feet to each of the 351 beds contained in the hospital.

The Westminster Hospital has a total site area of 27,500 feet, contains 205 beds, so that the site area per bed is 134 feet.

The Royal Free Hospital has a total site area of 40,455 feet which gives a site area of 253 feet for each bed.

The Middlesex Hospital has a total site area of 81,947 feet, and contains 307 beds, so that the area per bed is 267 feet.

St. Mary's Hospital, on its original site, had a site area of 38,280 feet and contained 281 beds, which gives an area of 136 feet per bed. An addition to the site has been acquired, upon which new wards and other buildings are being erected, but, like St. George's Hospital, the site area per bed is never likely to exceed 200 feet.

At Charing Cross Hospital the site area is 15,150 feet; it contains 175 beds, so that the site area per bed is only 86 feet. In this case the figures may be slightly varied owing to recent reconstruction and extension, but I have not the later figures by me.

The University College Hospital is in process of rebuilding. The total site area is about 55,526 feet. When completed it is to contain 400 beds, which would give an area of about 160 feet per bed. King's is in course of removal, and I do not know that the precise area of its new site has been mentioned or the proposed number of beds decided on. It will thus be seen that none of the smaller hospitals and medical schools have an area of anything like 300, much less of 450 superficial feet per bed.

THE FOUR GREATER LONDON GENERAL HOSPITALS WITH MEDICAL SCHOOLS.

I will next pass on to consider the position of the four larger general hospitals with important medical schools attached to them. The main site of the London Hospital is self-contained, being entirely surrounded by streets. Additional land has been purchased of recent years across the street upon which further new buildings have been erected. The site on which the hospital proper stands contains 240,327 feet, has accommodation for 776 beds, showing an area per bed of 310 feet.

Guy's hospital has a site area of 237,103 feet; it has accommodation for 652 beds and so contains an area of about 364 feet per bed. This site has been increased by the purchase of land to the north of the Inquest Room and Mortuary, in addition to the separate site upon which the residential college has been erected at the corner of Maze Pond.

St. Thomas's Hospital.—The site of this hospital has, on one side, the River Thames, throughout its whole length, and, on the opposite side, Palace Road, Lambeth. One extremity faces Westminster Bridge Road, and the opposite end of the site is narrowed off to a point by Palace Road. Nothing could be better than a site like this for a town hospital, except perhaps that its contiguity to the river may subject the wards to influences at certain seasons of the year which were best avoided. The total site area is 373,042 feet, which gives an area of 652 feet per bed for each of the 572 beds which the original buildings could accommodate.

St. Bartholomew's Hospital originally had a total site area of 203,690 feet. It provides accommodation for 674 beds, showing an area per bed of about 300 feet dead reckoning. It has recently purchased nearly two acres of land from Christ's Hospital's Trustees, which brings the total area up to 290,320 feet, and will represent an area of about 420 feet per bed dead reckoning, providing the total beds are brought up to 700, as is proposed. I have used the words "dead reckoning" in connection with this hospital because the site is so crowded with a number of buildings as I propose to show later.

Minimum Requirements in Superficial Feet and Beds.

I have thus far shown, mainly by figures, (1) that the site area of a large general hospital with a medical school attached should not be smaller than one which will provide at least 450 superficial feet per bed, and (2) that, with the exception of St. Thomas's Hospital, and probably Guy's Hospital, no London hospital with a medical school attached possesses a site which at present fulfils this essential requirement of modern science. Formerly 300 superficial feet per bed were regarded as enough for a town hospital; but the continuous increase in the demands for special departments, and, above all, for the complete aeration of every building occupied by patients, has produced results which raise the minimum of efficiency to 450 superficial feet per bed. In the case of a virgin site upon which it is possible to plan a hospital and medical school in its entirety, it is possible to so arrange the accommodation in the various buildings that every available foot of site shall be utilised to the best advantage. In London, owing to the circumstances I have already referred to, with the exception of St. Thomas's Hospital, the medical school buildings have been added from time to time, and were probably not contemplated when the hospital buildings were first planned and erected. This accounts, too, in a large measure, for the position in which two of the larger general hospitals stand with regard to this question. I have taken out the figures, and I find that upwards of 56 per cent. of one site is occupied by buildings, although the ward blocks, theatres, etc., occupy less than 16 per cent. of the whole available area, against about 20 per cent., or one-fifth, of the site which is covered by medical school and kindred buildings. I mention this to show that whereas for a town hospital the unit has been, roughly, 100 beds per acre of site, if such a hospital has a medical school attached to it, the unit must be increased to something like 50, or at the outside 60, beds per acre.

The University of London to the Rescue.

In these circumstances, knowing as I do the extraordinary difficulties which have had to be met by the authorities in the case of most of our great London general hospitals having medical schools attached, I welcome with satisfaction the scheme propounded by the Institute of Medical Sciences Committee of the University of London, which was adopted by the Senate in the spring of the present year. That scheme provides for common courses of instruction for internal medical students, not only in chemistry, biology, and physics, but in anatomy, physiology, and pharmacology, which will ultimately relieve the hospitals from the necessity of providing accommodation, appliances, and teachers for these subjects, as they have to do at the present. It will at the same time relieve them from a burdensome weight, and their whole energies can then be devoted to the teaching of the clinical subjects of medicine and surgery, which in all their various branches have largely developed of late. It will have the further advantage of greatly improving the hygienic condition of the sites of most of the great London general hospitals, by freeing them from existing blocks of buildings, which will then cease to be necessary in connection with the medical schools.

It will give some idea of the enormous cost involved, and of the great services which these London hospitals have rendered to the Empire and nation through their medical schools, to mention that it is estimated that a sum of £375,000 will be required by the University of London to enable them to build and equip the various institutes of anatomy, physiology, biology, chemistry, and physics and endow the professors of these subjects. The gain to the students and to medical education in London will be most material, for there is no other city of the United Kingdom in which there are such opportunities for the study of medicine and surgery. Any person of large means who takes an intelligent interest in the development of London institutions, and especially in the field of science, has here presented to him a splendid opportunity for wisely expending some of his money by lending a hand to the University of London in support of the Institute of Medical Sciences, in connection with which a most attractive scheme has been approved by the Senate.

St. Bartholomew's Hospital: Its Present Position.

In speaking of the site area per bed of St. Bartholomew's Hospital, I qualified the figures given by adding the words "dead reckoning." It is essential, when considering the site area per bed a particular institution should possess, to clearly apprehend the whole of the buildings which are to be placed upon it. In fact, London has been placed at a great disadvantage with regard to its medical school buildings, from the circumstance that the hospitals were in most cases first erected, and that subsequently medical school buildings have been placed upon some of these sites to an extent which cannot be justified if due regard is to be paid to the hygienic requirements of a modern hospital.

Sir Henry then proceeded to point out on the plans exhibited the drawbacks of the Mansion House scheme, and the advantages of the proposed alternative on the 8-acre site, explaining that while the latter demonstrated that all that was required could be provided for, and was the result of much experience and careful thought, yet it was not put forward as a hard and fast plan from which no departure could be made. The Mansion House scheme retained the old wards of 160 years ago, and by interference with their light and air would lessen still further their efficiency. The nurses' home would be noisy and altogether unsuitable, for it was to be placed along Little Britain, a narrow thoroughfare through which during practically most of each 24 hours a constant stream of heavy traffic passed. Financially, the alternative plan which he advocated had this great advantage, that the existing buildings could be utilised for the nursing home, the residential college for students, and other purposes, while to pull them down would cost a huge sum, since they were very solidly built with walls six feet thick. If the Mansion House scheme were proceeded with St. Bartholomew's was doomed, and a great national, a great Imperial asset, would be lost. It was inconceivable that the City of London would be content with a hospital that would be practically dead in 25 years. What the City of London required, and should have, was a great and efficient general hospital, up-to-date in every respect and on an adequate site—the most perfect institution that the most knowledgeable and able brains could devise. In conclusion, he hoped he had said nothing that could give offence to anyone in talking about superficial feet, bricks and mortar, light and air.

---

The Chairman said they had listened to a remarkably able address that had given them all something to think about and talk about. As regards St. Bartholomew's he confessed to being in sympathy with Sir Henry, and cordially agreeing with his views. It would be most regrettable if the present opportunity were to be wasted. He invited discussion on the general question of hospital sites.

Mr. Conrad Thies, Secretary of the Royal Free Hospital, said the best compliment they could pay Sir Henry for his very admirable paper was to express their own views. With hospitals in London they must take into account the character of the surrounding buildings. He agreed that it would be a thousand pities if the reconstruction of Bart's was not carried out on the best possible plan. At the same time his idea was that they should follow the example of the Continental countries in building hospitals in the country. In Paris, just now, the great Hôtel Dieu was being built out in the suburbs. Medical progress was more and more making it clear that the sick should be treated in the purest air possible. Looking at the plans for St. Bartholomew's, it seemed to him they must be wrong to build a great institution to draw people requiring medical and surgical treatment into an atmosphere breathed by six million people. He was convinced the sick poor should be treated on the outskirts of cities. They had object lessons abroad. At Hamburg there was a hospital built on the outskirts where the results to the sick poor had quite come up to expectations. Building hospitals in the middle of London was building them on the most valuable land in the world, and he thought there should be a small receiving hospital in the City and the larger institution out in the country. Not only from the point of view of the patients was it important to have good air, but from that of the nurses and staff. The amount of sickness among the nursing staffs of the hospitals was a source of great anxiety; even when they got away from duty the fresh air was too far to get at. He looked to the time when London would have a series of receiving hospitals and out-patient departments, while the bulk of the patients were treated in the country.

The Chairman said the question of site was a most important one, and agreed that, looked at superficially, it certainly seemed that to move out into the country was the best thing that could be done. But they must remember there were many points to consider, not quite obvious at first sight. Patients suffering from acute diseases or injuries were not in a condition to travel far. There was also the question of teaching, and there would be great difficulty with respect to the students. They could not be in London and in the country as well, and if much time were taken in going to and fro, it must interfere with the teaching. So far as treatment is concerned, no doubt the country had great advantages. Those hospitals which had convalescent homes could draft out the cases that were settling down toward recovery, and so make room for more acute cases. But even for the patients the students were a great advantage for these young acute minds, eager to follow and criticise everything kept everybody up to the mark.

Mr. Jonathan Hutchinson, F.R.S., consulting surgeon to the London Hospital, said that both the chairman and himself were old enough to remember the time when St. Thomas's was removed from close to Guy's to its present position. At that time he was connected with the press, and he did his best to get St. Thomas's taken out into the country. Since that time he had had much experience in hospital work, and was familiar with all sorts of opinions on the subject, but he had not in the least changed his own. He thought that many of those who knew the cost of that institution would agree with him that it was a mistake to build St. Thomas's in its present position. He was strongly of opinion that it was to the advantage of patients to be treated in country air. They were really only on the threshold of the knowledge of what country air really meant. Fresh air and sunlight were of paramount importance as healing agencies, and he believed that future generations would look on ourselves as benighted with regard to this question. Until they could build hospitals with all the wards looking south and near the ground, so that there was free access of sunlight and air to the patients and of the patients to the sunlight and fresh air, so long should he be an opponent of great hospitals in towns. Of course they must provide in the towns for cases of accident and emergency and for severe operations, but a great number of cases—scrofulous cases, chronic diseases, children, could be taken right out into the country which in itself would be superior to any other treatment. He had in his mind by way of illustration the case of a boy he had dealt with at the London Hospital. The lad was playing with a box of matches he was carrying in his trousers pocket, set light to them and got an awful burn right in the groin. He treated him for a long time and tried everything, but he could not get the wound to heal. Then he sent him to Margate and in a few weeks the boy was back quite well. Nature's great tonics were sunlight and fresh air, and it would be of the greatest benefit after an operation or a bad illness if just at the turning point the patient could be wheeled out into the open. Of course, no hard and fast rule could be laid down, but he thought it would be desirable if each of the large hospitals could divide into two in that way. With the increased facilities for transit which were becoming available he thought the difficulties of distance would be greatly lessened. He would not recommend the erection of great and substantial buildings. Let them be put up cheaply, of wood or canvas, spread out over a large area, as they could be where land was cheap, so that the patients could be carried out into the open without trouble. There would be a great saving in that method of building, and there would be no need to run them up to great heights. Then turning to the out-patient departments. He was sure it was not wise to build the very large out-patient rooms that were being erected; the gigantic crowds of patients were attended with very great inconvenience. Let the staff do their very best, it was almost impossible to attend properly to them all. He would suggest that they should affiliate with the dispensaries in the neighbourhood. Let them be strictly affiliated, and officered by medical men from the hospitals themselves. They would then have the advantage that the medical officer would be able to send suitable cases off to the hospital at once while other cases could be seen at their homes as much as possible. This would economise the time of the patients, which was a great consideration for them, especially those with children. He thought this system would be an advance in medical education. Students could be alloted to each dispensary and required to attend at regular hours, and so learn punctuality and responsibility in the discharge of duty and come into closer touch with their teachers. Of course it might be said that if the out-patient department were brought too close to the patients it would be to the loss of the medical practitioners in the neighbourhood, but he thought it would be no more difficult to prevent abuse in small institutions near the patients' homes than now—in fact it would be more convenient to make inquiries. The officers could be encouraged to be on good terms with the medical men of the neighbourhood, they could aid in diagnosis and persuade the people to be treated at home. One other suggestion he should like to make: he thought the medical schools should be separated from the hospitals. There was no need for them to occupy the same buildings. The hospital could then be kept for clinical teaching and the schools for the study of science and preliminary subjects. The teachers and professors, too, should be paid not by the students' fees but by salaries from the institutions to which they were attached. It would be a good thing if some one large hospital would admit, not students, but those who had passed their examinations. Further, he thought that the reputation of London would be advantaged if a number of hospitals would amalgamate so far as their schools were concerned, and so do away with competition, and allow their students to attend at several. He was sure such an arrangement would have good results, and care was certainly needed, for they had very active competitors both across the Atlantic and in the North.

Mr. Timothy Holmes, Treasurer of St. George's Hospital, said he should like to say a word on the subject of the advantages of having a hospital in the country for operations. They had had experience at St. George's, for they had a country branch—not only a convalescent home, but a hospital where they could treat anything. It was given them about forty years ago by an old student who left a quarter of a million. They thought that operations would be far better carried out in the country air, and he had a most acute remembrance of the transaction. It was no joke to have to go down every day for a week or a fortnight. He allowed that the comfort or convenience of the doctor ought not to weigh as against the advantage of the patient—but that was just the point. The patient did not recover any better. They were surprised to find that the operations at St. George's were just as successful; he did not say those at the country branch were worse, but they were no better, and accordingly, taking the other considerations into account, they gave up the scheme. He was convinced that for such cases as are generally taken in—serious operations, such as amputations or ovariotomy—the advantages of the country had been exceedingly exaggerated. And of course, at the time he was speaking of, hospitals were suffering from erysipelas and pyæmia and such-like troubles from which they were now happily delivered. As to the question of St. Bartholomew's he had come to hear Sir Henry Burdett's opinion with regard to the two plans, and he was not sufficiently acquainted with the facts to care to express an opinion of his own.

Sir Henry Burdett, referring to the points raised during the discussion, said he thought that the putting of hospitals outside the towns altogether was a counsel of perfection. The question of site was really one of accessibility more than anything else. Patients would go to the institution in which they had most confidence, and that was not altogether a matter of the reputation of the staff because often the particular doctor was accessible at some other institution much nearer to the patients' homes than the hospital to which they came. If they considered a big city like London which was extending every year, they must ask where were they going to take the hospitals to? Wherever they went, if the hospital was to be at all within access—even if they built a special systems of electric trams—in a few years' time it would be surrounded with houses, for the population of London was growing at the rate of 80,000—the population of a big town—every year. In practice it was not really a question of putting the hospitals in the town or outside it, but of having attached to the hospitals branch hospitals in the country which would be links to the town hospitals to which the latter could send patients when conditions admitted. They must still have, in the town, hospitals built on an adequate site area. From the financial point of view the cost of the town site was not really material, for if they gave a quarter of a million for a site they would have a piece of land that would possibly be worth half a million when they wanted to sell. But the cost of land acted as an estoppel to extension, and if someone, having made a fortune, would devote his money to giving hospitals more adequate sites, he would be doing the greatest possible good with his wealth. He would not only do an immensity of service to poor people who must be treated within reach of their own homes, but would have a memorial greater than that to be obtained by the establishment of many free libraries. They had had the testimony of three consulting surgeons—from Guy's, the London, and St. George's—and the effect of their testimony was that they must have hospitals in London each with a branch hospital in the country. The town hospital must be the original reception house where cases could be diagnosed and dealt with and then passed into the fresh air so soon as practicable to expedite recovery. They should avoid great expensive buildings where possible—for instance, in some cases, instead of adding a new wing to a hospital, they should establish a branch in the country, and so relieve the pressure on their wards. Mr. Thies had referred to the Plenum system washed air, but he confessed he was not constructed to live under a pressure of so many pounds to the square inch, and at an even and unvarying temperature. As to the cleanliness of the washed air, he had been along the flues through which it went and found, as the state of the walls and ceilings often proved, that the air so admitted was far from clean. He had gone into the question with Dr. John Billings, who built the Johns Hopkins Hospital, and they both agreed this system was not needed or suited to a temperate climate. It was often most trying for the staff to live in a hospital worked on the Plenum system. Referring to what Mr. Jonathan Hutchinson had said about cheap buildings, it had been demonstrated that, in tuberculous cases, in big buildings, even though quite open, with no windows at all, a patient's temperature would often not go down but when he was moved to a châlet, it fell to nearly normal within 48 hours and kept there; and this in spite of the nervousness felt by women, especially under the latter conditions. Even these timid people recognised the improvement in their condition and felt so much better that they would not afterwards leave the cheap châlet for the big costly building. He instanced also the Moabite Hospital in Berlin with 700 beds, built temporarily at a cost of £10,000, for fever cases, but now and for many years occupied as a general hospital, and answering as well as if it had cost hundreds of thousands.

A vote of thanks moved by Sir Edmund Currie to the Governors of Charing Cross Hospital for the use of the room, and one to the Chairman closed the proceedings.

